# Reversible swelling of SBMV is associated with reversible disordering

**DOI:** 10.1016/j.jsb.2017.06.003

**Published:** 2017-12

**Authors:** Jade Li, Carl Fricks, Ivan Rayment, Donald L.D. Caspar

**Affiliations:** Rosenstiel Basic Medical Sciences Research Center, Brandeis University, Waltham, MA 02254, USA

**Keywords:** Southern bean mosaic virus, Icosahedral surface lattice, Reversible swelling, Disorder, Modeling, Dynamic, X-ray diffraction, Proton magnetic resonance

## Abstract

The structures of the compact and swollen southern bean mosaic virus (SBMV) particles have been compared by X-ray diffraction and proton magnetic resonance (PMR). Small-angle X-ray scattering showed that removal of divalent cations at alkaline pH causes the particle diameter to increase from 289 Å in the native SBMV by 12% in solution and by 9% in microcrystals. The swelling is fully reversible upon re-addition of Ca^2+^ and Mg^2+^ ions, as shown by the X-ray patterns at 6 Å resolution and by the 270 MHz PMR spectra. Beyond 30 Å resolution, X-ray patterns from the compact SBMV in solution and in microcrystals show fine fringes of ∼1/225 Å^−1^ width extending to 6 Å resolution, whereas patterns from the swollen SBMV in solution and in microcrystals show only broader fringes of ∼1/90 Å^−1^ width, Model calculations demonstrate that the fine fringes from compact SBMV arise from regular packing of the protein subunits on the icosahedral surface lattice; the smearing of fine fringes in the swollen virus pattern can be simulated by uncorrelated displacements of pentamers and hexamers of protein subunits, with a standard deviation of 6 Å from their mean locations. The PMR spectrum of compact SBMV is poorly resolved, whereas PMR spectrum of swollen SBMV shows sharp resonances in the methyl proton region. The line-narrowing for a fraction of the aliphatic protons upon swelling cannot be accounted for by rotational relaxation of the particle of 6 × 10^6^ MW, but must be attributed to internal motion in small regions of the protein subunits.

## 1 Introduction

Southern bean mosaic virus (SBMV) is an isometric particle of 6.6 × 10^6^ Daltons (Yphantis, 1964). It comprises a protein capsid, built from 180 identical subunits of 29,000 molecular weight (Tremaine, 1966, Ghabrial et al., 1967) arranged in a T = 3 icosahedral surface lattice (Caspar and Klug, 1962), and the encapsulated RNA (Rutgers et al., 1980). Atomic absorption spectra of the native particle suggest that divalent metal ions are bound in stoichiometric amounts to each protein subunit (Hsu et al., 1976, Hull, 1978). EDTA treatment renders the particle measurably free of divalent cations and causes it to swell reversibly at slightly alkaline pH (Hsu et al., 1976, Hull, 1977a, Rayment et al., 1979). An increase of Stokes radius by nearly 13% was inferred from the lowering of sedimentation coefficient from 115 S to 100 S (Wells and Sisler, 1969, Brown and Hull, 1973, Hsu et al., 1976, Hsu et al., 1977). Re-addition of Ca^2+^ and Mg^2+^ ions in stoichiometric amounts allows the swollen particle to re-contract to the compact, 115 S form; however omission of either ion results in contraction to intermediate sizes (Hsu et al., 1976). In contrast to the compact particle, swollen SBMV is susceptible to disassembly by sodium dodecyl sulfate and by high ionic strength, as well as to cleavages by several proteases and ribonucleases (Sehgal and Sinha, 1974, Hsu et al., 1976, Hull, 1977b). Such differences in Stokes radius and stability between the compact and swollen particles indicate significant changes in conformation and interactions among the subunits.

The pH-dependent swelling is a general property of isometric plant viruses. Since the first report by Incardona and Kaesberg (1964) on swelling of brome grass mosaic virus, similar phenomena have been studied in this (Incardona et al., 1973, Chauvin et al., 1978) and other RNA viruses, including SBMV, the cow pea chlorotic mottle (Bancroft et al., 1967, Adolph, 1975, Jacrot, 1975), turnip rosette, sowbane mosaic, carnation mottle, rice yellow (Hull, 1977a) and tomato bushy stunt (TBSV; Robinson and Harrison, 1982) viruses, as well as a DNA virus, cauliflower mosaic virus (Chauvin et al., 1979, Ani et al., 1979). These viruses differ in the extent of swelling and of re-contraction. Divalent metal ions are generally found to stabilize the compact form, or to remove hysteresis in the back titration to the compact form (Jacrot, 1975, Chauvin et al., 1978). These effects, and that the alkaline pH required for swelling coincides with the dissociation of the carboxyl-carboxylate ion, prompted the suggestion (Bancroft, 1970) that a pair of carboxyl groups on adjacent protein subunits are bridged by a divalent cation, and the absence of this cation leads to charge repulsion at alkaline pH and particle swelling. The swollen form has been proposed as an intermediate in viral assembly or disassembly under physiological conditions (Pfeiffer and Durham, 1977, Durham et al., 1977).

Swelling of highly symmetrical macromolecular assemblies such as isometric virus particles requires cooperative structural transformations. The subunit interactions in the compact state of TBSV (Harrison et al., 1978) and of SBMV (Abad-Zapatero et al., 1980) have been elucidated to atomic resolution by X-ray crystallography. Extensive homology of polypeptide chain folding was demonstrated between the SBMV subunit and the shell domain (S-domain) of the TBSV subunit, which are primarily β-barrels. TBSV has an additional projecting domain (P-domain). Extensive contacts between these globular domains in neighboring subunits, and intertwining of N-terminal arms from one third of the subunits appear to provide cohesion in these capsids. The crystal structure of swollen TBSV has been solved to 8 Å resolution using the structure of compact TBSV as starting model (Robinson and Harrison, 1982). The result shows that the inter-subunit contacts within pentamer and hexamer clusters are conserved upon swelling, but contacts around the quasi-threefold positions are disrupted. In native TBSV, a pair of EDTA-chelatable cations (most likely Ca^2+^) is located on the boundary between S-domains related by the quasi-threefold axis (Hogle et al., 1983). They bridge five aspartate side chains from neighboring S-domains, and their chelation at alkaline pH results in charge repulsion between the deprotonated aspartates, hence rupture of the trimer contacts (Robinson and Harrison, 1982). In native SBMV, a Ca^2+^ ion is bound at an equivalent site between the quasi-threefold related subunits, to two aspartate side chains from one subunit and the carboxyl terminus from the neighbor (Silva and Rossmann, 1987; Fig. S1). Therefore swelling of the divalent-cation free SBMV at alkaline pH (Hsu et al., 1976, Hull, 1977a) can be expected to involve rupture of the trimer contacts while conserving the pentamer and hexamer clusterings.

Swollen SBMV was first crystallized by Rayment et al. (1979). The crystals grown at pH 6 are approximately isomorphous with the orthorhombic Type III crystals of the native (Akimoto et al., 1975), but show a 2% increase in each cell dimension. Alternatively, by treating native SBMV in the rhombohedral Type II crystal lattice with EDTA in the presence of 25% ethylene glycol at pH 8, a 2% increase of cell constant was also induced. In the mother liquor surrounding the treated crystals, a 7% increase of effective scattering diameter was observed by X-ray solution scattering (Rayment et al., 1979). Crystals of swollen SBMV diffract X-rays to 4 Å resolution whereas those of compact SBMV diffract to at least 2.8 Å resolution (Abad-Zapatero et al., 1980), indicating disorder in the swollen crystals. Decreased maximum resolution was also observed from crystals of the TBSV swollen by about 10% as compared to those of the compact TBSV (Robinson and Harrison, 1982).

We have compared X-ray patterns in the small-angle region, and extending to 6 Å resolution, of compact and swollen SBMV that are in solution and in microcrystals. These conditions allow us to observe the spherically averaged total intensity. This total intensity includes contributions from variations in the structure, in addition to diffraction from the averaged structure which is that sampled by the Bragg reflections. Our analysis indicates that there is substantial disorder within the swollen SBMV particle. The dynamics of this disorder have been investigated using proton magnetic resonance (Li et al., 1982) and ^13^C and ^31^P nuclear magnetic resonance (McCain et al., 1982a, McCain et al., 1982b).

## 2 Materials and methods

### 2.1 Virus preparation

The southern bean mosaic virus stock (cow pea strain) was kindly provided by Dr. J. E. Johnson of Purdue University. The virus was grown in cowpea plants and was isolated in the native, compact state using a modification (Johnson et al., 1974) of the purification procedure of Ghabrial et al. (1967).

The virus structure was converted from the compact into the swollen form at slightly alkaline pH following the removal of divalent metal ions, using an extension of the procedure of Rayment et al. (1979): The compact virus at a concentration of 20 mg/ml was dialyzed at 4 °C against deionized water for 12 h, and then against 50 mM Na phosphate (pH 7.5) containing 40 mM EDTA for 24 h. These treatments had been found to remove Ca^2+^ and Mg^2+^ ions to less than one part in 10^6^ (Rayment et al., 1979). Prolonged contact of virions with EDTA was avoided by another dialysis against 50 mM phosphate buffer (pH 7.5) for 12 h at 4 °C. 1 mM NaN3 was present in all dialysis solutions.

The swollen virus was re-contracted by dialysis against a solution of 20 mM MgCl_2_, 12 mM CaCl_2_ and 1 mM NiCl_2_. The re-contracted virus was then transferred by dialysis to the same buffer (250 mM Na phosphate, pH 7.0) as the native, compact preparation.

For X-ray solution scattering experiments, the compact, swollen, and re-contracted virus preparations were concentrated by sedimentation at 100,000 × *g*_max_ (24,000 rpm in Beckman SW27 rotor) for 1 h. The pellets were resuspended to approximately twice their volume using the phosphate buffer, at 250 mM (pH 7.0) for the compact and re-contracted preparations, and at 50 mM (pH 7.5) for the swollen. Different buffer concentrations were used here, because the compact virus crystallizes at low ionic strength, whereas the swollen virus is unstable at high ionic strength. The final virus concentration was determined by 0.D._260_ of aliquots diluted 1000-fold. This concentration was about 120 mg/ml.

Microcrystals of both the compact and the swollen virus particles were obtained by mixing a virus solution at 15 mg/ml in 50 mM phosphate buffer (pH 7.5) with an equal volume of 16% (w/v) PEG, *M*_r_ = 6000, in the same buffer. The microcrystals were allowed to sediment by gravity in the X-ray capillary and excess liquid was removed before the diffraction experiments.

### 2.2 X-ray diffraction

For each type of virus preparation, diffraction patterns of either the same specimen or identically treated specimens have been collected in both the small-angle region (*S* < 0.025 Å^−1^) and the high-angle region (to 0.15 Å^−1^), using two X-ray cameras. The small-angle diffraction patterns were recorded at a specimen-to-film distance of about 410 mm. The Cu-Kα radiation from an Elliott GX13 rotating anode was focused by a bent mirror and a quartz monochromator set at right angle to it. The full-width-at-half-maximum of the focused beam is 80 µ in the direction of the monochromator, corresponding to an order-to-order resolution of 1.2 × 10^−4^ Å^−1^ (∼1/8000 Å). The high-angle diffraction patterns extending to 0.15 Å^−1^ reciprocal spacing were recorded using X-rays from an Elliott GX6 rotating anode and a specimen-to-film distance of about 120 mm. The X-rays were focused by two perpendicular mirrors, and the order-to-order resolution was 1.1 × 10^−3^ Å^−1^ (∼1/900 Å).

### 2.3 X-ray data processing

The X-ray films were microdensitometered on an Optronics P-1000 Photoscan. Diffraction patterns with the high order-to-order resolution were scanned using a 25 µ raster and those with the lower order-to-order resolution, using a 50 µ raster. From the former patterns, the observed intensity at a given reciprocal spacing was obtained by averaging optical densities along concentric arcs within a 15° sector about the direction of maximum order-to-order resolution, *i.e.* the direction of the monochromator. From the latter patterns, which were taken on the double mirror camera, optical densities were circularly averaged.

In the small-angle region, to obtain the diffraction amplitudes from the virus in solution, a smooth background through the intensity minima was subtracted from the measured intensity and the square root of the background-subtracted intensity was phased according to the transform of a uniform density sphere of equivalent diameter. Approximation of the small angle diffraction by the transform of an equivalent sphere has been found to be satisfactory for initiating the phasing of the crystallographic data of SBMV in the compact state, to 22.5 Å resolution (Johnson et al., 1976). The structure factors for the microcrystals were obtained, following background subtraction as above, by correcting the integrated intensities of the powder lines for the Lorentz factor and the multiplicity factor of the lattice. For powder lines observed at the same reciprocal spacing S, the integrated intensity was divided equally between them. The structure factors were phased by comparison with the diffraction amplitudes from solutions of the virions in the same state.

### 2.4 Model calculations

At reciprocal spacings up to 0.02 Å^−1^, the diffraction pattern is dominated by the spherically symmetric component of the virus particle transform. By comparing the measured diffraction amplitude in this region against the transform *F*_sphere_ (*S*) of a uniform density sphere of radius *r* (F_sphere_ (*S*) *=* 2 *r*^2^ *j*_1_(*2πSr*)/*S*, where *S* = 2 sin θ/λ is the reciprocal spacing, and *j*_1_ is the spherical Bessel function of first order), one can determine the radius of the equivalent sphere corresponding to the virus particle.

Non-spherically symmetric components of the density distribution in the structure contribute to the diffraction fringes at larger reciprocal spacings. These components of the density distribution arise from the icosahedral arrangement of the 180 subunits of the virus coat protein on a *T* = 3 surface lattice (Caspar and Klug, 1962), and from the asymmetric structure of the protein subunits themselves and of the RNA. A simple model for the distribution of scattering matter in the virus particle consists of a set of 180 point densities on an icosahedral surface lattice, plus a spherically symmetric inner core (Jack and Harrison, 1975). The contribution of the 180-point surface lattice model to the spherically averaged intensity is the spherical transform of the distribution function of scalar lengths joining pairs of points on the surface lattice (Guinier and Fournet, 1955). That is,

$$I(S)=\underset{i}{\sum }\underset{j}{\sum }f_{i}f_{j}[\text{sin}(2\pi \;S\;r_{\mathrm{ij}})/2\pi \;S\;r_{\mathrm{ij}}]\text{,}$$

where *f_i_* and *f_j_* are the structure factors of the *i*^th^ and *j*^th^ scattering elements, which for point elements have the value of unity, *r_ij_* is the distance between these points. The calculation of this scattering length distribution function is simplified by the icosahedral symmetry: The 180-subunit, *T* = 3 structure can be represented as 12 regular pentamers centered on the 5-fold axes and 20 regular hexamers centered on the 3-fold axes. The scattering length distribution of the entire lattice is the sum of the distribution of scalar lengths from one subunit in a pentamer to all the subunits, multiplied by 60, plus the scalar length distribution from one subunit in a hexamer to all the subunits, multiplied by 120.

### 2.5 Proton magnetic resonance spectroscopy

Virus solutions for proton magnetic resonance (PMR) experiments were prepared as in Section 2.1, except that H_2_O in the phosphate buffers were replaced by D_2_0, either by dialysis against the D_2_0 buffer, or by repeated centrifugation and resuspension in the D_2_0 buffer. The PMR spectra were recorded by Dr. A. G. Redfield of Brandeis University on a 270 MHz Fourier transform spectrometer. Small-angle X-ray scattering patterns of the same preparations were taken to determine the swelling of the virions in D_2_0.

## 3 Results

### 3.1 Small-angle scattering from virus in solution

**Fig. 1:**
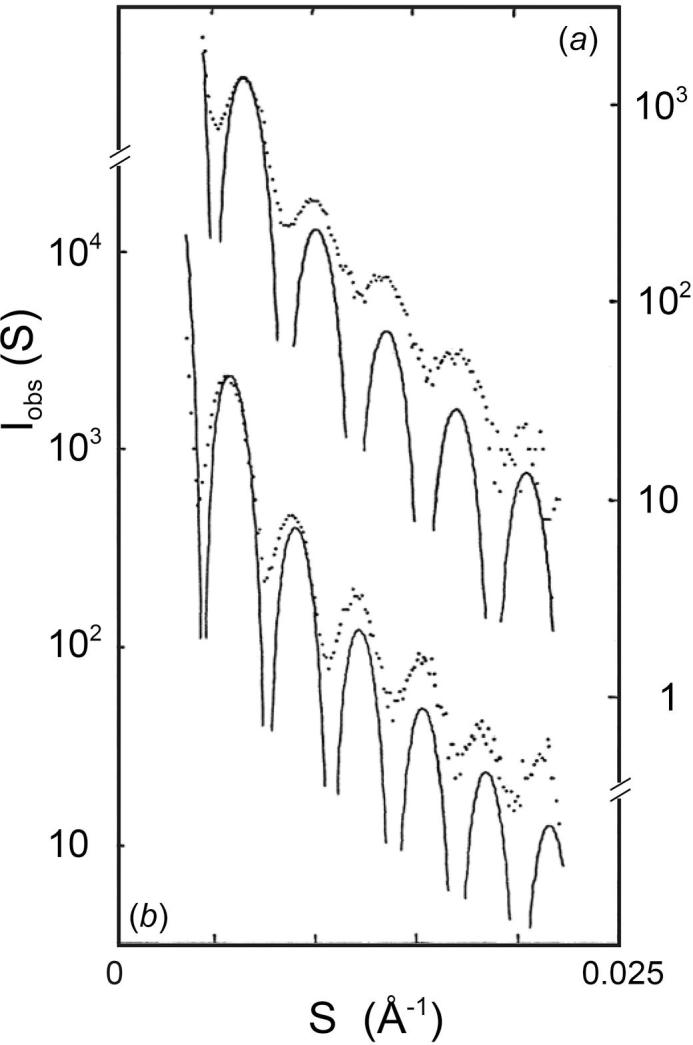
Small-angle scattering from SBMV particles in solution (····) and the intensities calculated for uniform density spheres of equivalent diameter (—): (*a*) Compact SBMV in 250 mM phosphate (pH 7.5), spheres of 289 Å diameter; (*b*) EDTA-treated SBMV in 50 mM phosphate (pH 7.5), spheres of 325 Å diameter. The fringe positions in the observed intensities are well accounted for by those in the transform of a uniform density sphere. Thus from the shifts in position of the fringes, the increase of particle diameter in solution, induced by removal of divalent cations at slightly alkaline pH, is determined to be 12%.

The small-angle region (*S* < 0.025 Å^−1^) of diffraction patterns has been recorded at an order-to-order resolution of 1.2 × 10^−4^ Å^−1^. The spacings on different patterns were determined with an accuracy of 0.5% relative to one another, by exposing the series of specimens under identical camera settings. Up to eight fringes can be distinguished in the small-angle pattern from the compact and swollen virions (Fig. 1). The positions of the first five fringes in the scattering curve of the compact virion correspond to those calculated for a uniform density sphere of diameter 289 ± 1.5 Å (Fig.1*a*). Diffraction patterns from the EDTA-treated virus show a similar series of fringes, which are more closely spaced and corresponded to the scattering from spheres of diameter 325 ± 1.6 Å (Fig.1*b*). Thus, the mean diameter of the SBMV particles at slightly alkaline pH is increased by about 12% following the removing divalent metal ions. The swelling was fully reversed by the re-addition of both Mg^2+^ and Ca^2+^ ions by dialysis. The diffraction pattern from the re-contracted virus was indistinguishable from that of the untreated, compact virus.

**Fig. 2:**
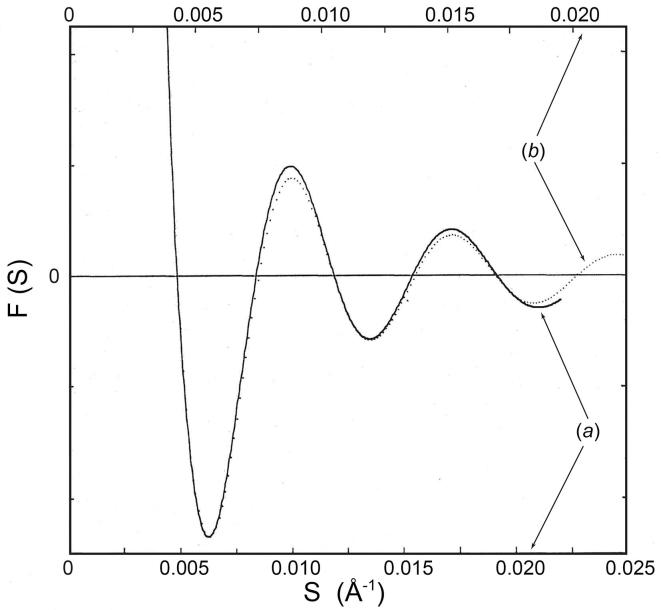
Comparison of the spherically averaged scattering amplitudes from (*a*) compact (—) and (*b*) swollen (····) SBMV particles in solution. The amplitudes are obtained by phasing the square root of the background-subtracted intensities in the small-angle region, according to the transforms of equivalent diameter spheres. In order to compare the shape of the amplitudes, the two curves have been scaled vertically to match the first subsidiary maxima, and horizontally to align the successive nodes. Relative to the first and third fringes, the second, fourth and fifth fringes are weaker in the amplitudes from the swollen particle than in those from the compact.

The spherically averaged scattering amplitudes of the virus particle were obtained from the solution scattering patterns. In Fig. 2 the amplitudes of the compact and swollen particles are compared, after expanding the reciprocal distance scale (upper abscissa) of the transform of the swollen particle by 12% relative to that of the compact (lower abscissa) to bring the first five fringes of the two amplitude curves into alignment. Besides confirming the 12% swelling, the alignment makes it clear that the shape of the scattering amplitude is different for the compact and swollen particles. Relative to their first and third subsidiary maxima, the swollen particle transform is weaker in the second, fourth and fifth fringes than the compact particle transform. These small but distinct differences in the spherically averaged particle transform show that the swelling of SBMV is not simply an isotropic expansion of the native compact structure. Because of the different ionic strengths required for the compact and swollen virus solutions, the solvent electron densities were different in the compact and swollen specimens, being 0.344 e/Å^3^ and 0.336 e/Å^3^ respectively; the observed changes in the diffraction amplitudes, however, cannot be accounted for by the change in contrast density alone. In an isotropic expansion, the difference between the two spherically averaged transforms, after adjusting the reciprocal spacing of the swollen pattern to compensate for the expansion, would be the transform of a sphere of uniform density equal to the contrast difference. The observed amplitude differences do not match this description. The observed differences are also unlikely to result from a change in solvent binding accompanying the change of ionic strength, because single crystal diffraction experiments on the closely related TBSV have found no change in solvent binding when the virus was placed in media of widely different electron densities ranging between 0.343 and 0.408 e/Å^3^ (Harrison, 1969). Therefore the observed change in the spherically averaged transform (Fig. 2) indicates changes in the radial electron density distribution in the virion.

### 3.2 Powder patterns from microcrystals

**Fig. 3:**
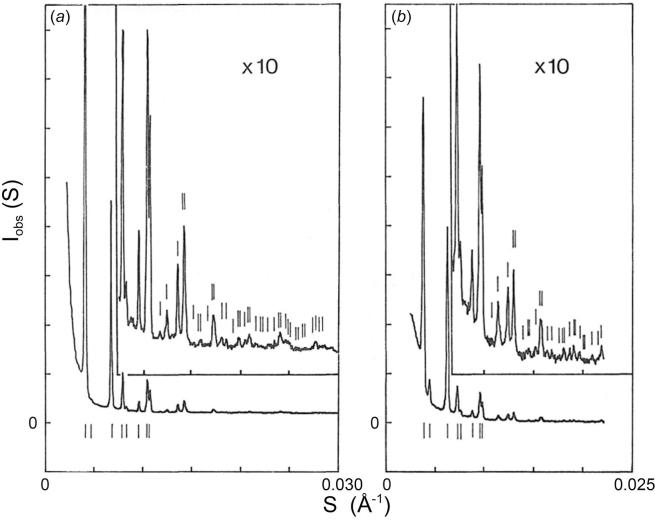
Powder patterns from microcrystals of (*a*) compact and (*b*) swollen SBMV taken on a small-angle camera having an order-to-order resolution of ∼1/8000 Å. The powder lines (line positions marked by small vertical bars) in both patterns index on face-centered cubic lattices where the lattice constant is 416.0 ± 2.0 Å for the compact and 454.5 ± 1.7 Å for the swollen, respectively. This shows a lattice expansion of 9%, corresponding to an increase in the nearest neighbor distance, from 294.1 ± 1.4 Å between compact particles to 321.4 ± 1.2 Å between the swollen.

Powder patterns from microcrystals of both the compact and swollen virions, obtained by precipitation from 8% (w/v) PEG 6000, were recorded on a camera with an order-to-order resolution of 1.2 × 10^−4^ Å^−1^ (∼1/8000 Å). The densitometer tracings are shown in Fig. 3. The powder lines in both patterns index on face-centered cubic (FCC) lattices (Table 1), with *a* = 4l6.0 ± 2.0 Å for the lattice of the compact virion and *a* = 454.5 ± 1.7 Å for that of the swollen. This lattice expansion corresponds to a 9% increase of the nearest neighbor distance, from 294.1 ± 1.4 Å between compact particles to 321.4 ± 1.2 Å between the swollen.

**Table 1:** Powder line spacings and structure factors for the face-centered cubic microcrystals.§

		Compact SBMV: *a* = 416.0 (2.0) Å			Swollen SBMV: *a* = 454.5 (1.7) Å		
*h k 1*		*S*_calc_	*S*_obs_	|*F(hkl)*|	*S*_calc_	*S*_obs_	|*F*(*hkl*)|
1 1 1		0.00416	0.00413	32.2	0.00381	0.00383	18.3
2 0 0		0.00481	0.00479	1.9	0.00440	0.00423	6.3
2 2 0		0.00680	0.00680	17.7	0.00622	0.00628	20.1
3 1 1		0.00797	0.00798	6.8	0.00730	0.00731	6.8
2 2 2		0.00833	0.00833	4.7	0.00762	0.00758	4.9
4 0 0		0.00962	0.00963	8.5	0.00880	0.00881	8.1
3 3 1		0.01048	0.01049	9.4	0.00959	0.00960	8.7
4 2 0		0.01075	0.01077	6.4	0.00984	0.00983	5.7
4 2 2		0.01178	0.01183	1.8	0.01078	0.01082	1.5
3 3 3	}	0.01249	0.01254	3.1	0.01143	0.01145	4.2
5 1 1							
4 4 0		0.01360	0.01360	7.5	0.01245	0.01244	7.0
5 3 1		0.01422	0.01427	5.3	0.01302	0.01303	3.6
4 4 2	}	0.01442	0.01442	3.3	0.01320	0.01303	3.6
6 0 0							
6 2 0		0.01520	0.01533	1.8	0.01392	0.01402	1.5
5 3 3		0.01576	0.01584	1.6	0.01443	0.01434	2.3
6 2 2		0.01594	0.01599	2.1	0.01459	0.01453	2.5
4 4 4		0.01665	0.01670	2.5	0.01524	0.01521	4.2
5 5 1	}	0.01717	0.01725	3.0	0.01571	0.01572	2.8
7 1 1							
6 4 0		0.01733	0.01729	4.2	0.01586	0.01584	4.7
6 4 2		0.01799	0.01808	2.8	0.01646	0.01643	1.9
5 5 3	}	0.01846	0.01855	1.6	0.01690	0.01686	2.1
7 3 1							
8 0 0		0.01923	0.01926	3.7	0.01760	0.01765	5.3
7 3 3		0.01968	0.01977	2.7	0.01801	0.01809	2.4
6 4 4	}	0.01982	0.02028	0.5	0.01814	0.01809	2.4
8 2 0							
8 2 2		0.02040	0.02048	3.0	0.01867	0.01872	3.3
5 5 5	}	0.02082	0.02087	2.3	0.01905	0.01908	2.4
7 5 1							
6 6 2		0.02096	0.02087	2.3	0.01918	0.01908	2.4
8 4 0		0.02150	0.02150	2.2	0.01968	0.01971	3.2
7 5 3	}	0.02190	0.02170	1.2	0.02004	0.02002	0.7
9 1 1							
8 4 2		0.02203	0.02213	1.7	0.02016	0.02022	0.7
6 6 4		0.02255	0.02232	1.7	0.02043	0.02035	0.7
9 3 1		0.02293	0.02287	2.1	0.02099	0.02101	3.0
8 4 4		0.02355	0.02346	2.0	0.02156	0.02168	3.4
7 5 5	}	0.02392	0.02394	1.6	0.02189	0.02192	2.5
9 3 3							
8 6 0	}	0.02404	0.02405	3.3	0.02200	0.02192	2.5
10 0 0							

§ *S* = 2sin θ/λ is the line spacing in Å^−1^. *S_hkl_* = √(*h*^2^+*k*^2^+*l*^2^)/*a* for FCC lattices. The lattice constant, *a,* is averaged from the observed spacings, *S_obs_*, and shown with standard deviation in parenthesis. *S*_calc_ is obtained from *hkl* and *a*. |*F*(*hkl*)| is the root-mean-square structure factor amplitude derived from the integrated intensity at radius *S_hkl_*. The values have been scaled to the corresponding continuous transform and signs have been assigned as shown in Fig. 4. The estimated uncertainties in the mean structure factor measurements are indicated by the error bars in Fig. 4.

### 3.3 Comparison of small-angle solution scattering patterns with powder patterns

**Fig. 4:**
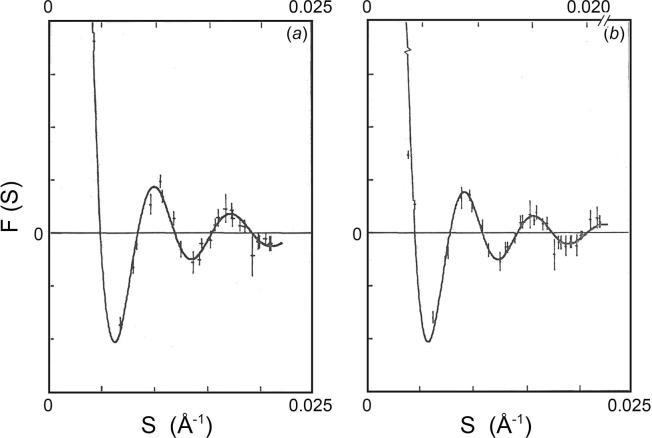
Comparison of the structure factors obtained from the microcrystal diffraction patterns with the spherically averaged scattering amplitudes measured in solution: (*a*) compact SBMV, (*b*) swollen SBMV. In each case, the sampled transform of the particle in the crystal is closely approximated by the spherically averaged particle transform, except where contributions from the non-spherically symmetric components are significant. In (*a*), the positions of nodes in the sampled transform show that the compact SBMV has the same particle diameter in microcrystals as in solution. In (*b*), fitting the sampled transform of the swollen particle in microcrystals to the continuous transform of the swollen particle in solution required a 3% expansion of the latter along the reciprocal distance axis (top abscissa). Therefore, the particle in microcrystals is 9% swollen and not 12% swollen as in solution.

In the small-angle region, structure factors for the compact and swollen virus particles in the FCC lattices have been obtained from the integrated intensities of the powder lines. In Fig. 4 these structure factors, representing the sampled transform of the particle in the lattices, were compared with the spherically averaged transform of the particle in solution. Their agreement reflects the dominance of the spherically symmetric terms in the particle transform at low resolution. The small discrepancies over some of the structure factors are evidence of the contribution from the non-spherically symmetric components, which have been averaged out in the formation of the spherically averaged transform.

From the position of the nodes in the sampled transform, the compact virus is found to have the same particle diameter in the microcrystals as in solution (Fig.4*a*); however, the sampled transform of the swollen virus in microcrystals corresponds to a particle diameter that is 3% smaller than the swollen particle in solution (Fig.4*b*). The 9% swelling of the EDTA-treated virus particle in the crystal, indicated by the sampled transform, is consistent with the 9% increase of the lattice constant compared to that of the compact virus.

### 3.4 Comparison of solution and microcrystal patterns at higher angles

**Fig. 5:**
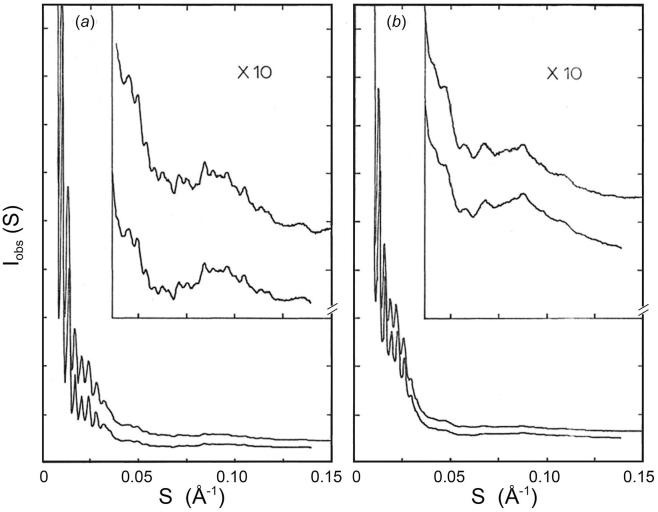
Comparison of diffraction patterns extending to high-angles, of (*a*) compact, and (*b*) swollen SBMV, in solution (upper curves) and in microcrystals (lower curves). The X-ray camera for these experiments has an order-to-order resolution of ∼1/900 Å, hence the lattice sampling seen in the small-angle patterns of microcrystals (Fig. 3) is not fully resolved. At higher angles, lattice sampling is so dense that the spherically averaged intensities in the microcrystal patterns correspond closely to the spherically averaged scattering from particles in solution. Beyond 0.035 Å^−1^ spacing, patterns of the compact SBMV in solution and in microcrystals are both characterized by high-frequency (1/225 Å) modulations, but patterns of the swollen SBMV, whether in solution or in microcrystals show only broad modulations of 1/90 Å. However there is a general similarity between the intensity envelopes in (*a*) and in (*b*) over these spacings.

**Fig. 6:**
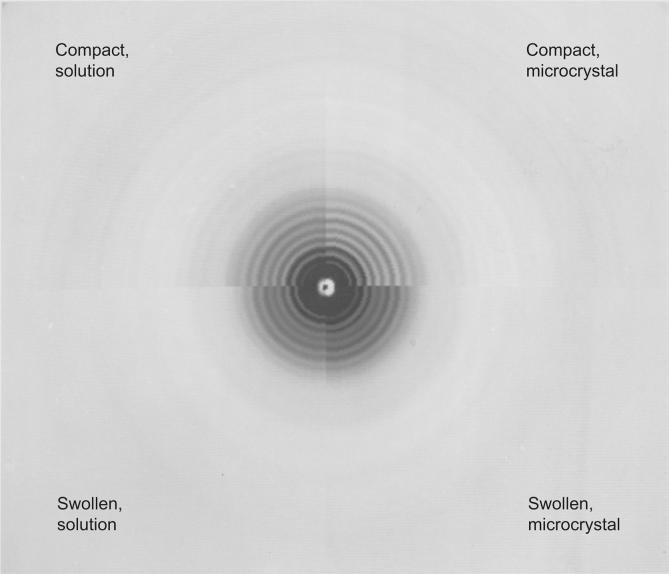
Composite of four diffraction patterns displayed to 0.125 Å^−1^ spacing on the Grinnell graphics terminal, from compact and swollen SBMV in solution and in microcrystals, as labeled. The mid-level of intensity display in this image has been adjusted to enhance contrast in the low-intensity range. Comparison of left and right quadrants demonstrates the similarity between solution and microcrystal patterns at high angles. The small-angle difference in fringe spacings that indicate a difference in the mean particle diameter of swollen SBMV in solution and in microcrystals is not accompanied by detectable high-angle intensity differences. Comparison of upper and lower quadrants in the small-angle region gives evidence for the increase of particle diameter, and in the high-angle region emphasizes the loss of high-frequency modulations upon particle swelling.

Diffraction patterns from the compact and swollen SBMV both in solution and in microcrystals have been observed to 0.15 Å^−1^. Densitometer tracings of the four patterns are shown in Fig. 5. A detailed visual comparison of the intensity variations in these patterns is illustrated in Fig. 6. This representation of the diffraction data was created on a Grinnell graphics terminal by displaying in respective quadrants the circularly averaged optical densities on a log scale as a function of the reciprocal spacing out to 0.125 Å^−1^.

Outside the small-angle region, when the lattice sampling in the microcrystal patterns becomes dense, a striking correspondence in the position and intensity of the fringes is seen between the solution and microcrystal patterns of the compact virus (Fig5*a*). Likewise, between the solution and microcrystal patterns of the swollen virus, fringes in the high-angle region agree in detail (Fig.5*b*), and this in spite of the evidence in the small-angle data already mentioned, that the mean particle diameter of the swollen virus in the crystal is 3% smaller than that of the swollen form in solution (*i.e*. being 9% swollen with reference to the compact particle as compared to 12% swollen).

Between patterns of the compact SBMV and those of the swollen virus, striking differences are found beyond 0.035 Å^−1^: In the patterns of the compact virus (Fig.5*a*), high-frequency modulations of period about 1/225 Å are observed to the limit of measure diffraction at 0.15 Å^−1^. Instead of such fine fringes, the patterns of the swollen virus are characterized by broader fringes of widths about 1/90 Å (Fig.5*b*). It is important to point out that when the swelling was reversed by the re-addition of divalent cations, the diffraction patterns reverted to those characteristic of the compact virus, to the resolution limit of 0.15 Å^−1^. Therefore, the smearing out of fine fringes in the high-angle diffraction patterns reflects intrinsic properties of the swollen state, and it does not result from degradation or any other irreversible alteration of the particle structure.

Scattering vectors arising from the protein subunits can be divided into intra-subunit and inter-subunit vectors. The length distribution of intra-subunit vectors is determined by the internal structure of the subunits, while that of the inter-subunit vectors depends on the packing arrangement of subunits in forming the viral coat. The lengths of intra-subunit vectors are limited by the subunit diameter. This diameter would be about 36 Å if the subunits of SBMV with molecular weight of 29,000 were spherical. Indeed, the crystal structure of the compact SBMV (Abad-Zapatero et al., 1980) shows the protein subunits to be globular with a maximum dimension of about 40 Å, in the direction perpendicular to the viral surface.

Clearly, the ∼1/225 Å, high-frequency modulations seen in the high-angle patterns of the compact SBMV originate from the longer inter-subunit vectors. Because of the particle symmetry, the inter-subunit vectors will be clustered into classes of different lengths. The prominence of the 1/225 Å modulation occurs because the longest inter-subunit vectors between the subunits related by an icosahedral five-fold, or three-fold, axis are clustered into the length class which is somewhat shorter than the particle diameter. The high-frequency modulations in the patterns from compact SBMV (Fig.5*a*) indicate that the long inter-subunit vectors have a narrow distribution of lengths, reflecting the rigidity of the particle. Furthermore, the compact particle in solution must be as well ordered as in the crystal, to produce the same high-resolution detail in the diffraction pattern (Fig.5*a*). The absence of high-frequency modulations at high angles in the swollen SBMV pattern (Fig.5*b*) indicates that the long inter-subunit vectors have a broad distribution of lengths; *i.e*. subunit packing in the swollen particle is disordered.

Comparison between the high-angle patterns from compact and swollen SBMV reveal a similarity of the intensity envelope, if the high-frequency modulations that are present only in patterns of the compact virus are ignored. This similarity is consistent with a swollen structure in which the shorter inter-subunit vectors, *e. g*. those within a pentamer or hexamer cluster, have remained relatively invariant through the swelling and associated disorder. Thus the disordered packing would result from preferential disruption of selected inter-subunit contacts.

Because the high-frequency modulations are not correlated with the internal structure of the protein subunit, but are correlated with the symmetry-dependent placements of the subunits, they can be qualitatively interpreted in terms of a simple 180-point model of the subunit arrangement.

### 3.5 Model calculations for the high-angle region

**Fig. 7:**
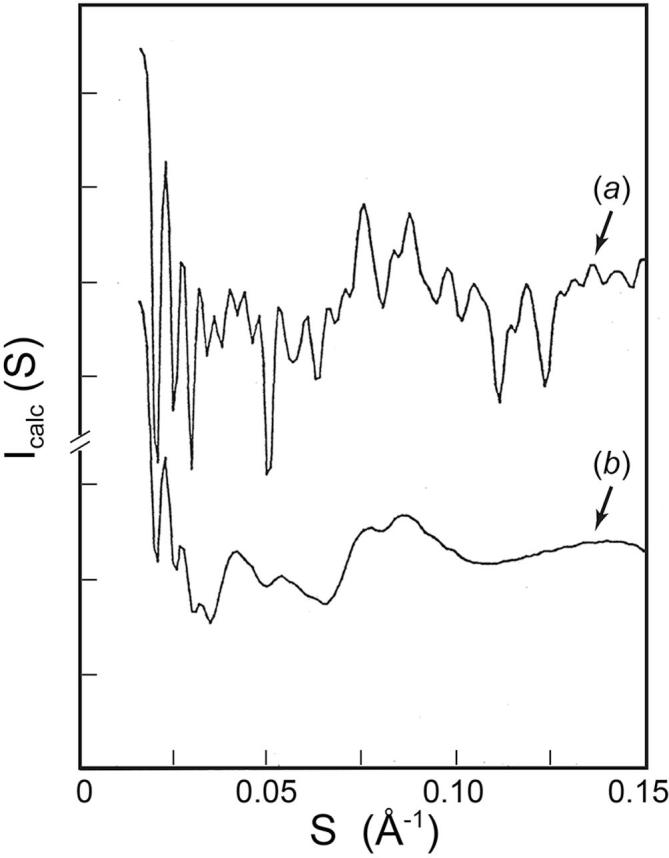
Spherically averaged intensities calculated from point models designed to represent subunit positions in the capsids of (*a*) compact and (*b*) swollen SBMV, respectively. The model for the compact SBMV consists of 180 points distributed in pentamer and hexamer clusters on a *T* = 3 icosahedral surface lattice (see text). The calculated intensity (*a*) simulated the high-frequency modulations of 1/225 Å width in the high angle patterns of compact SBMV (*cf*. Fig.5*a*), but not the more gradual intensity variations because the shape and internal structure of subunits are not represented by the point model. The model for the swollen SBMV allows each pentamer or hexamer cluster of points to be displaced radially as an invariant unit with a standard deviation of ∼6 Å from its mean radial position, uncorrelated with the displacements of any other clusters. The calculated intensity (*b*) simulated the loss of high-frequency modulation and the appearance of a 1/90 Å modulation in the high-angles patterns of swollen SBMV (*cf*. Fig.5*b*).

The point model for the compact SBMV was constructed to represent the arrangement of the most prominent density features visible in the 22.5 Å resolution map (Johnson et al., 1976). This map shows densities on the viral surface clustered around the 5-fold and 3-fold axes, with densities around the 5-fold located at higher radii than those around the 3-fold. In addition, the protein shell in compact SBMV is known to extend from a radius of 110 Å to 145 Å (Abad-Zapatero et al., 1980, Krüse et al., 1982). We distributed the 180 points in pentamer and hexamer clusters on the surface of a *T* = 3 icosadeltahedron. When the 60 points representing the 12 pentamers are located at 129 Å from the center of the icosadeltahedron and 15 Å from the fivefold axes, and the 120 points representing the 20 hexamers are at 116 Å from the center and 14 Å from the threefold axes, the spherically averaged intensity calculated from the point model (Fig.7*a*) shows high-frequency modulations with the average fringe width observed in the high-angle patterns of the compact virus (Fig.5*a*).

Since the point model does not represent the shape and internal structure of the subunits, the calculated intensities cannot be expected to reproduce the more gradual modulations in the observed intensity. However, the simulation demonstrates that the presence of high-frequency modulations in the high-angle region depends only on the ordered placement of the subunits on the icosahedral surface lattice. This provides a starting point for modeling the effects of disordered subunit packing on the spherically averaged diffraction pattern.

In diffraction patterns of the swollen SBMV, the fringes observed beyond 1/30 Å spacing have a width of about 1/90 Å (Fig.5*b*), indicating that clusters of subunits with outer dimension around 90 Å maintain an invariant structure to the resolution limit of the experiment. Comparison with dimensions in the compact SBMV (Silva and Rossmann, 1987, PDB code 4SBV) suggests that the invariant clusters are most likely to be the pentamers and hexamers. In the related TBSV, the pentamer and hexamer clusterings were conserved in the swollen state (Robinson and Harrison, 1982). Therefore in our model for the swollen SBMV particle we have chosen to keep invariant the inter-subunit vectors within the clusters of pentamers and hexamers.

The disorder was introduced into the point model by allowing the radial location of each cluster to fluctuate about its mean uncorrelated with the displacements of its neighbors. These displacements distort the icosahedral symmetry of the particle. However, the probability distribution of the radial positions was allowed to be the same for all pentamer clusters, and analogously for all hexamer clusters. The overall scattering vector distribution is given by 60 times the length distribution of vectors from a pentameric subunit, plus 120 times the length distribution from a hexmeric subunit. We assumed a Gaussian distribution for the radial position of each cluster about its mean. The distribution was sampled through two standard deviations of the displacement to each side of the mean, at intervals of one-tenth the standard deviation. Vectors between different clusters were weighted by the product of the Gaussian factors for the respective radial positions. Vectors within the same cluster were weighted once by the Gaussian factor for their radial position. It was found that a standard deviation of ∼6 Å in the radial displacements of the clusters from their mean locations led to the loss of high-frequency fringes outside 0.035 Å^−1^ and the appearance of a 1/90 Å fringe modulation (Fig.7*b*).

### 3.6 Proton magnetic resonance spectra

The X-ray evidence for disorder in the swollen SBMV cannot determine whether the disorder is static, due to the particles presenting a range of distortions from the icosahedrally symmetric mean structure, or dynamic, indicating mobility in flexible segments of the particle. To distinguish between these two possibilities, the proton magnetic resonance (PMR) spectra of compact and swollen SBMV were compared.

**Fig. 8:**
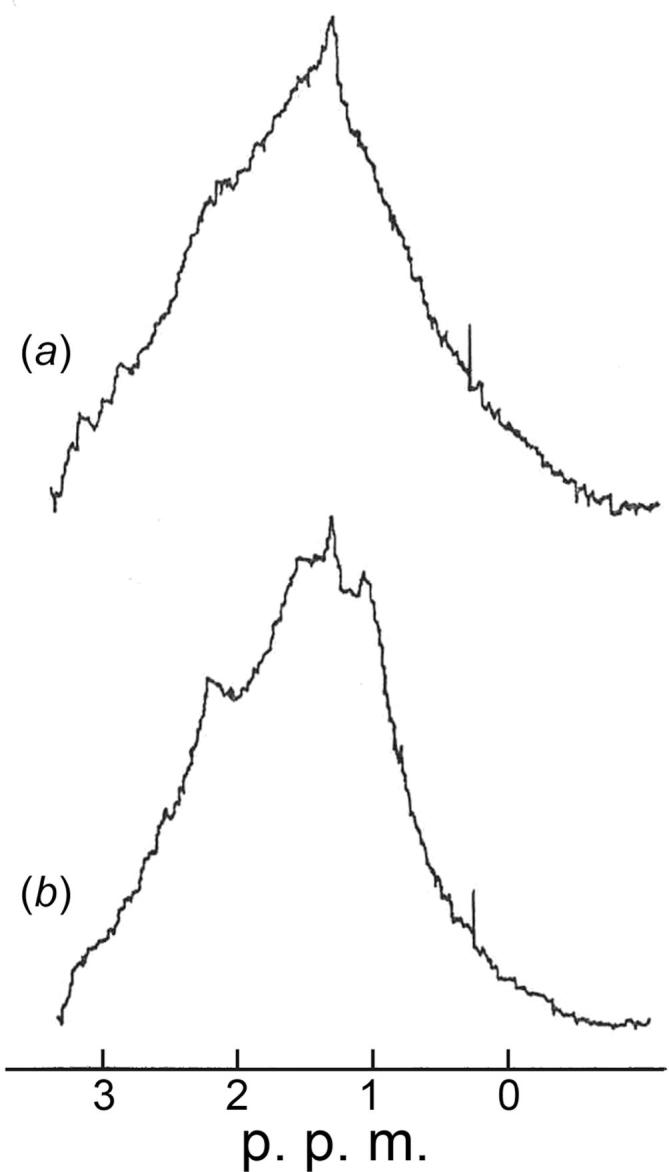
Comparison of the aliphatic region of the 270 MHz PMR spectrum from (*a*) compact and (*b*) swollen SBMV. The spectrum of the compact particle is broad as predicted from the slow rotational diffusion associated with the large molecular weight of 6.6 × 10^6^ Daltons. The spectrum of the swollen particle contains small, relatively sharp peaks in the methyl proton area, at *δ* = 1.07, 1.59 and 2.18 p. p. m. from 4, 4-dimethyl-4-silapentane-1-sulfonic acid (DSS), which indicate internal motions that are more rapid than the rotational diffusion of the swollen particle as a whole. The spectrum of the re-contracted particle is indistinguishable from that of the compact particle.

The 270 MHz PMR spectrum of the compact SBMV is poorly resolved in the aromatic region and also very broad in the aliphatic region (Fig.8*a*). This is not surprising given the large size of the particle (MW 6.6 × 10^6^). Raising the temperature from 26 to 45 °C failed to induce sharp components in the spectrum, indicating a rigidity in the compact state. When the virus is brought into the swollen state, as confirmed by the small-angle X-ray pattern of the sample in the D_2_O buffer, the aromatic spectrum is unchanged, but superimposed on the broad spectrum in the aliphatic region are small, relatively sharp peaks in the methyl proton area, at *δ* = 1.07, 1.59, and 2.18 p.p.m. downfield from DSS (Fig.8*b*). These peaks have been observed with the same relative intensity and sharpness in different swollen virus preparations, as well as after exhaustive dialysis. Therefore they are not due to small molecular weight contaminants, nor due to diffusible degradation products from the virus particle.

These observations show that a line-narrowing for a fraction of the aliphatic protons, to an experimental linewidth of 10–20 Hz, is associated with the swelling. Since instrumental conditions were identical for the spectra from compact and swollen viruses, the line-narrowing is attributed to differential relaxation effects in the two samples. By evaluating the rotational correlation time *T*_r_ for SBMV using the relation, *T*_r_ = 4π*ηa*^3^/3*kT* (Wüthrich, 1976), where *a* is the particle radius proportional to the molecular weight, *η* is the viscosity of the medium, and *T* is the absolute temperature, it can be shown that a rigid sphere model of relaxation cannot account for the observed sharp PMR components (*cf*. Jardetzky et al., 1978). Rotational diffusion would contribute less to line-narrowing for the swollen particle than for the compact, since the 12% increase of diameter leads to a 30% decrease of tumbling rate. Therefore the observation of sharp PMR signals indicates significant internal motion in the swollen SBMV particle, involving aliphatic groups.

Two other observations show that the internal motion is characteristic of the swollen state. Firstly, the PMR spectrum of SBMV re-contracted by the addition of both Ca^2+^ and Mg^2+^ ions is indistinguishable from that of the native, compact virus. Thus the mobility associated with swelling is also reversible. Secondly, when the procedure for swelling was abbreviated to an 8-h dialysis of the compact virus in 250 mM phosphate (pH 7.5) against 40 mM EDTA in the same buffer, followed by dialysis against the 50 mM phosphate (pH 7.5), the preparation obtained was not swollen based on the criterion of small-angle X-ray scattering, and it had a PMR spectrum typical of the compact SBMV. However, heating this preparation to 45 °C caused small peaks to appear in the PMR spectrum at the same frequencies as seen at room temperature from the SBMV swollen by the standard procedure. Similar heating had no effect on the spectrum of the untreated, compact virus. The temperature dependence in the treated sample suggests that these sharp signals arise from thermal motion, which in turn is controlled by the removal of divalent metal ions.

## 4 Discussion

### 4.1 Extent of swelling

We have measured the extent of swelling in divalent cation-free SBMV at pH 7.5 compared with the compact virus particle at pH 7.0, in solution and in a FCC crystal lattice, using a small-angle X-ray camera with an order-to-order resolution of (1/8000 Å). In solution, the effective particle diameter is defined by fitting the Fourier transform of a uniform density sphere to the spherically averaged particle transform derived from the background-corrected small angle scattering pattern out to the fifth subsidiary fringe (Fig. 1). The effective particle diameter was found to be 325 ± 1.6 Å for the swollen virus compared with 289 ± 1.5 Å for the compact, indicating a 12% swelling in solution. In addition, we compared the spherically averaged particle transforms of the compact and swollen virus directly. A 12% expansion of the swollen particles transform along the reciprocal distance axis brought the first five fringes of the two transforms into alignment (Fig. 2), which demonstrated the 12% swelling in solution without reference to specific models. In the FCC crystal lattice, effective particle diameter is estimated in two ways: (1) fitting the measured particle transform sampled by the crystal lattice to the first five fringes of the spherically averaged transform measured in solution; (2) measuring the inter-particle separation in the lattice (Table 1). For the compact SBMV, the effective particle diameter in the FCC lattice is equal to that in solution (Fig.4*a*). For the swollen SBMV, the effective particle diameter in the FCC lattice found by fitting transforms was 316 ± 1.6 Å, corresponding to a 9% increase from the compact rather than 12% increase as in solution (Fig.4*b*). Since the increase in inter-particle distance is likewise 9% (Table 1), the smaller effective particle diameter suggests some lattice constraint.

The extent of swelling of SBMV reported under different conditions has been somewhat varied but generally smaller than we found. In the rhombohedral type II crystal treated with EDTA at pH 8 in the presence of ethylene glycol, the lattice constant increased by 2% but the effective scattering diameter measured from sampled transform increased by 4%, while scattering diameter in the mother liquor swelled by 6.5% (Rayment et al., 1979). The fact that the lattice constant increases less than the scattering diameter was proposed to show that the five-fold vertex, where the particles are in contact, protruded less prominently on the swollen particle than on the compact. The lesser swelling in the Type II lattice than in mother liquor is similar to our observation of the smaller 9% swelling in the FCC lattice compared with 12% in solution, although crystal contacts in the FCC lattice are along the two-fold axes of the particle. Krüse et al. (1982), fitting the first three subsidiary maxima of their neutron scattering data measured at three different D_2_O/H_2_O contrast levels to concentric shell models of RNA-protein distribution, inferred 10% swelling. In a related note, sedimentation coefficients of 100 S (Hsu et al., 1976), 107 S (Krüse et al., 1982), 98 S and 73 S (Wells and Sisler, 1969) have been observed in swollen SBMV, whereas for compact SBMV it is consistently 114–115 S (Wells and Sisler, 1969, Hsu et al., 1976, Krüse et al., 1982). It is not clear why different procedures for removing divalent cations have led to different degrees of swelling, but it is evident that relatively homogeneous swollen preparations can be obtained that show different mean diameters under slightly different conditions.

The 12% swelling of SBMV observed in solution using our procedure is reproducible and is fully reversible by the re-addition of Ca^2+^ and Mg^2+^ ions. Small-angle patterns of the re-contracted virion, whether in solution or microcrystals, are indistinguishable from those of the native compact virion. This reversibility is in agreement with the hydrodynamic data of Hsu et al. (1976).

In the transition from the compact to the swollen state, small changes in the shape of the first five fringes of the spherically averaged scattering amplitudes (Fig. 2) indicated a change in the radial electron density distribution. This reorganization that is detected at very low resolution must involve relative displacements of rather large domains in the virion, although the magnitude of net change in the spherically averaged density distribution is small. The neutron scattering data measured at different D_2_O/H_2_O contrast levels indicated a change in the relative radial distribution of protein and RNA on swelling (Krüse et al., 1982).

### 4.2 Disordered subunit packing in the swollen particle

The most striking difference between X-ray patterns of the compact and swollen SBMV is observed beyond 30 Å resolution, where most of the diffraction is due to the non-spherically symmetric components of the density distribution. The intensities from the compact virus in solution and in microcrystals are modulated by the same set of fine fringes of 1/225 Å average spacing. The patterns of the swollen virus, whether in solution or in microcrystals, present only broad fringes of widths 1/90 Å or greater.

High-frequency modulations correspond to long scattering vectors in the virion, which must be inter-subunit and not intra-subunit vectors. The high-frequency modulations of the high-angle intensity signify the presence, in the Patterson function, of strong peaks at large distances. This characteristic of the Patterson function (synonymous with the length distribution of scattering vectors) is a consequence of the high symmetry of the icosahedral surface lattice. Therefore, the high-angle data indicates that the symmetry of subunit packing is rigidly maintained in the particle of the compact SBMV. Moreover, the compact particle must be as well ordered in solution as in the microcrystals to give rise to the same set of high-frequency modulations. The intrinsic order of the compact particle is evident in the rhombohedral Type II lattice that diffracts coherently to at least 2.8 Å resolution (Abad-Zapatero et al., 1980).

The absence of high-frequency modulations in the high-angle pattern of swollen SBMV indicates disordered subunit packing, which broadens the length distributions of inter-subunit vectors, particularly those at large distances. Since the smearing of fine fringes is observed over the same scattering angles whether the swollen particles are in solution or in microcrystals, the disorder affecting subunit packing must be similar in magnitude in the two environments. It is interesting that the 3% difference in mean diameter of swollen particle in the crystal compared with that in solution, evident in the small-angle diffraction, does not lead to detectable differences in the higher angle diffraction (Fig. 5 and Fig. 6). The most probable explanation is that the disorder in the swollen particles is fairly large, so that at high angles subunit clusters (pentamers and hexamers) diffract independently of each other.

Upon re-contraction of the swollen particle as determined from the small-angle data, the high-angle patterns of the solution and microcrystals are again modulated by fine fringes and are indistinguishable from those of the native SBMV. Therefore, the disordering of subunit packing is reversible, and it is an intrinsic property of the swollen particle.

### 4.3 Modeling the subunit packing in compact and swollen SBMV

We modeled the ordered subunit packing in the compact SBMV by an icosahedral arrangement of 180 points in pentamer and hexamer clusters. With the points distributed at the approximate locations of to the most prominent surface features in the 22.5 Å resolution map (Johnson et al., 1976), the calculated spherically averaged intensity simulated the high-frequency modulations with the same average fringe width as is observed in the high-angle patterns from compact SBMV. The calculated intensity was not expected to reproduce the more gradual intensity variations or the reciprocal spacing of observed local maxima, because the point model does not describe the shape and internal structure of the subunits. However the point model simulation does illustrate that the high-frequency modulations originate from the rigidity of the compact SBMV, which gives long-range order over the surface lattice resulting in tight length distributions for the longest inter-subunit vectors. The high multiplicity under icosahedral symmetry contributes to the prominence of the high-frequency modulations.

The loss of high-frequency modulations in the high-angle diffraction from swollen SBMV can come about in two ways: either the icosahedrally symmetry is conserved with a heterogeneity in diameter, or the diameter is homogeneous (see Section 4.1) but there is internal disorder that broadens the length distribution expected from strict icosahedral symmetry. In solution scattering experiments, the measured intensity is the transform of the length distribution in the population, and the same degree of smearing results from the same lengths variability. Therefore solution measurements cannot distinguish between length variation among different particles and length variation within individual particle due to deviation from the icosahedrally symmetric “ideal”. We fortunately have observed high-angle diffraction from swollen SBMV in solution as well as in microcrystals. In both cases, the modulations of a 1/225 Å fringe width seen from compact SBMV are smeared out beyond about 25 Å resolution, which leads to an estimated length variability of around 5%. From small-angle region of the microcrystal diffraction, the inter-particle distances for both compact and swollen SBMV have been measured with accuracy within 0.5% using a small-angle X-ray camera with high order-to-order resolution. The mean diameter of the swollen particle in the FCC lattice is thus not more variable than that of the compact. Indeed if the diameter of swollen SBMV varied symmetrically by 5%, as required to smear out the fringes, then the swollen SBMV might not have formed crystals. Thus the loss of high-frequency modulations, in solution and in crystal, results not from variable particle diameter but from internal disorder in the swollen particle. For the swollen particle to be accommodated in the lattice and show internal disorder, one would expect the icosahedral symmetry of the particle to be distorted locally.

We modeled the disorder in swollen SBMV by uncorrelated radial displacements of invariant subunit clusters from their mean location in the icosahedral surface lattice. The invariant clusters are most likely pentamers and hexamers, rather than trimers, dimers, monomers, or any non-symmetric oligomers. A Gaussian standard deviation of about 6 Å in the radial positions of the clusters was found to explain the qualitative character of the observed high-angle diffraction from swollen SBMV, including loss of high-frequency modulation and appearance of a broader 1/90 Å modulation. This magnitude of positional variation corresponds to an isotropic *B*-factor of ∼950 Å^2^, indicating significant disorder in the swollen particle. This explains why the same smeared high-angle diffraction is observed from swollen SBMV in solution and in microcrystals, despite their 3% difference in diameter.

Our model for the disordered subunit packing in the swollen SBMV, which holds the pentamers and hexamers invariant, attributes the length variations for the long inter-subunit vectors to a preferential weakening of the trimer interactions at the quasi-3-fold positions. That a Ca^2+^ ion, which in the compact SBMV is bound between the quasi-threefold related subunits and is ligated to carboxyl groups from the neighboring subunits (Silva and Rossmann, 1987; Fig. S1), must be removed before swelling can occur at slightly alkaline pH (Hsu et al., 1976), supports the possibility that it is primarily the trimer contacts that are disrupted in the swelling process, as has been observed in the swelling of TBSV (Robinson and Harrison, 1982).

### 4.4 Motional character of the disorder in swollen SBMV

X-ray evidence for disorder in the swollen particle does not distinguish between static and dynamic disorder. Therefore proton magnetic resonance data were obtained in parallel. The reversible swelling of SBMV is correlated with a reversible sharpening of a number of the aliphatic proton resonance peaks. Because of its large molecular weight, rotational diffusion of the virion as a rigid sphere predicts very broad PMR spectral lines. The observed spectra of compact and re-contracted SBMV, and the aromatic region of the spectrum of the swollen SBMV, are featureless as expected from very broad overlapping signals. The appearance of sharp peaks from the swollen particles therefore indicates the occurrence of internal motion that is rapid compared to the rate of rotational diffusion of the virion as a whole. The sharp signals are small and superimposed on an otherwise very broad spectrum. Thus the mobile segments comprise a small fraction of the protons in the swollen particle.

Proton magnetic resonance evidence for internal motion has been established for the V segment of the coat protein of tobacco mosaic virus in the absence of RNA (Jardetzky et al., 1978). The aromatic region of the PMR spectrum is poorly resolved, even when the TMV protein is in the 4 S form (average molecular weight 52,000). The aliphatic region in the absence of RNA shows sharp peaks, some of which persist as the molecular weight is increased by polymerization into large helical aggregates, just as some sharp peaks are observed from the swollen SBMV here. The peaks from the TMV protein have been shown to originate from residues in rapid motion and have been assigned to the V segment by comparative studies of coat protein mutants. The binding of RNA to form the intact TMV particle abolishes this set of sharp signals. The relative magnitudes of the sharp peaks and broad background observed from the TMV coat protein in the RNA-free helix are comparable to those observed from the swollen SBMV particle. The similarity of the SBMV and TMV data suggests that the sharp PMR signals observed from swollen SBMV arise from the mobility of relatively short segments of the coat protein. ^13^C-NMR study of SBMV by McCain et al. (1982a) also showed increased mobility in selected amino acid residues upon swelling. ^31^P-NMR data indicated that the RNA core enters a mobile, solvated phase in the fully swollen SBMV particle (McCain et al., 1982b).

Analysis of our X-ray results indicates that in the swollen SBMV particle clusters of subunits are displaced from their mean locations with a standard deviation of 6 Å. At the same time, the PMR data suggest mobility in small segments of the protein. Taken together, the X-ray and PMR evidence leads to a picture of the disorder that can be best described by a mechanical analog. The viral capsid may be regarded as a system of masses connected by springs (*cf*. Peticolas, 1979): The clusters of subunits seen to be displaced as a unit in the X-ray analysis stand for the masses, and the boundary regions between them contain the springs. The X-ray analysis characterized the relative displacements of the subunit clusters, because of their proportionally large scattering power. The PMR experiments detected motions in the flexible regions. When the virus particle swells, certain contacts between the clusters are disrupted and the boundary regions have greater flexibility. The subunit clusters consequently adopt more variable positions (see animation of swelling model with flexible links between pentamer and haxamer clusters, SBMV_swelling_animation_lg.gif).

SBMV_swelling_animation_lg.gif
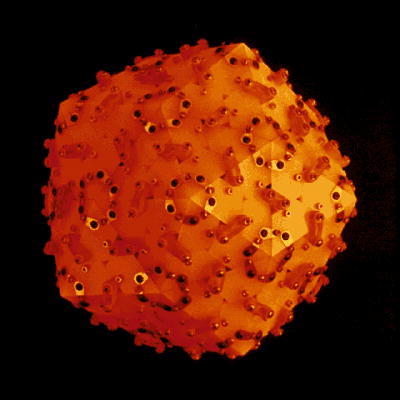

The variable mass distribution is detected by X-ray diffraction as disordered packing. Therefore, the mobility detected by PMR and the disorder detected by X-ray diffraction are complementary manifestations of the same physical process. Our findings imply that macromolecules are not homogeneous, but comprise parts of differing mechanical properties, some of which are capable of latent mobility.

## Appendix A Supplementary data

Supplementary Fig. S1 Main picture shows one icosahedral asymmetric unit bound by the five-fold axis at its top vertex and two three-fold axes at the bottom vertices. The trimer of subunits A, B, and C from the asymmetric unit of compact SBMV is superposed by secondary-structure-matching on its counterpart from compact TBSV, resulting in an *rmsd* = 1.47 Å for 154 aligned Cα atoms. The P-domain of TBSV is omitted for clarity. In SBMV, a Ca^2+^ ion (colored yellow) is bound on each of the quasi-threefold related subunit boundaries; its location corresponds to one cation in the pair (colored beige) bound on the equivalent boundary in TBSV. Insets compare the amino acid ligands for the divalent cations on the C-A subunit boundary of the two viruses. Superposing the bound Ca^2+^ ion in SBMV on the corresponding Ca^2+^ ion in TBSV causes the amino acid ligands Asp138, and Asp141 from the C-subunit and Asn259 from the A-subunit in SBMV to be superposed respectively on Asp183, Asp186 and Asp225 from the corresponding subunits in TBSV. Residues Asp138 and Asp141 in SBMV are equivalent by structural-based sequence alignment to residues Asp183 and Asp186, respectively, in TBSV; the C-terminal residue Leu260 in SBMV corresponds to the start of the P-domain in TBSV.

SBMV_swelling_animation_lg.gif
