# Supplementary figures and images for: Reversible swelling of SBMV is associated with reversible disordering
Source: J Struct Biol. 2017 Dec;200(3):314–24. doi: 10.1016/j.jsb.2017.06.003 (PMC5784231; doi:10.1016/j.jsb.2017.06.003)

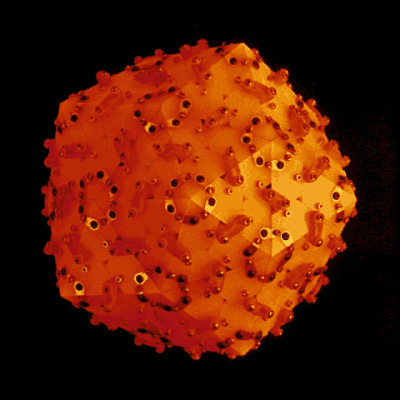

Supplement: SBMV_swelling_animation_lg.gif [file mmc2.gif]
