# Supplementary material for: Reversible swelling of SBMV is associated with reversible disordering
Source: J Struct Biol. 2017 Dec;200(3):314–24. doi: 10.1016/j.jsb.2017.06.003 (PMC5784231; doi:10.1016/j.jsb.2017.06.003)
Supplement: Supplementary Fig. S1 — Main picture shows one icosahedral asymmetric unit bound by the five-fold axis at its top vertex and two three-fold axes at the bottom vertices. The trimer of subunits A, B, and C from the asymmetric unit of compact SBMV is superposed by secondary-structure-matching on its counterpart from compact TBSV, resulting in an rmsd = 1.47 Å for 154 aligned Cα atoms. The P-domain of TBSV is omitted for clarity. In SBMV, a Ca2+ ion (colored yellow) is bound on each of the quasi-threefold related subunit boundaries; its location corresponds to one cation in the pair (colored beige) bound on the equivalent boundary in TBSV. Insets compare the amino acid ligands for the divalent cations on the C-A subunit boundary of the two viruses. Superposing the bound Ca2+ ion in SBMV on the corresponding Ca2+ ion in TBSV causes the amino acid ligands Asp138, and Asp141 from the C-subunit and Asn259 from the A-subunit in SBMV to be superposed respectively on Asp183, Asp186 and Asp225 from the corresponding subunits in TBSV. Residues Asp138 and Asp141 in SBMV are equivalent by structural-based sequence alignment to residues Asp183 and Asp186, respectively, in TBSV; the C-terminal residue Leu260 in SBMV corresponds to the start of the P-domain in TBSV. [file mmc1.pdf]

Supplementary information.

Figure S1.

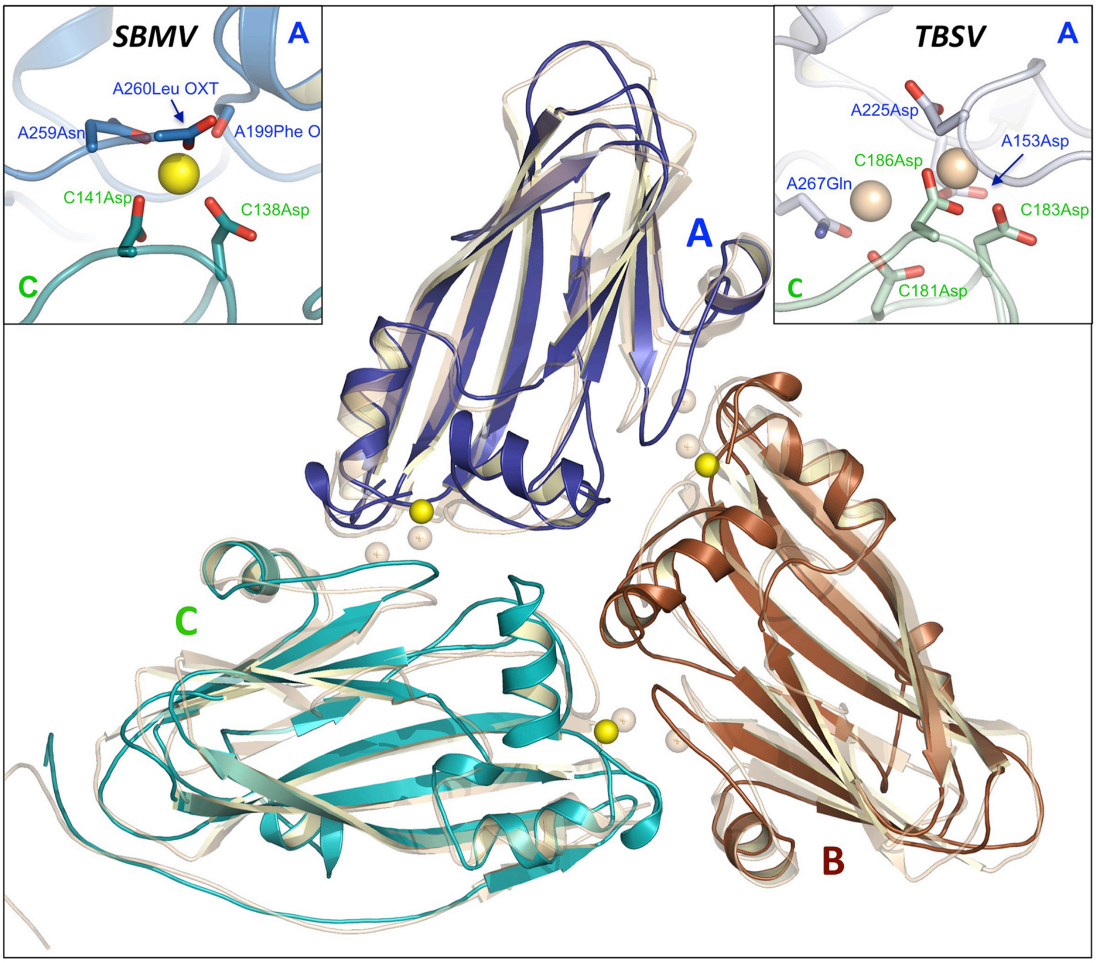

Figure S1. Similarity of the structural context of the Ca^{2+} binding sites in SBMV (PDB code 4SBV) and TBSV (PDB code 2TBV).

Main picture shows one icosahedral asymmetric unit bound by the five-fold axis at its top vertex and two three-fold axes at the bottom vertices. The trimer of subunits A, B, and C from the asymmetric unit of compact SBMV is superposed by secondary-structure-matching on its counterpart from compact TBSV, resulting in an *rmsd* = 1.47 Å for 154 aligned Cα atoms. The P-domain of TBSV is omitted for clarity. In SBMV, a Ca^{2+} ion (colored yellow) is bound on each of the quasi-threefold related subunit boundaries; its location corresponds to one cation in the pair (colored beige) bound on the equivalent boundary in TBSV. Insets compare the amino acid ligands for the divalent cations on the C-A subunit boundary of the two viruses. Superposing the bound Ca^{2+} ion in SBMV on the corresponding Ca^{2+} ion in TBSV causes the amino acid ligands Asp138, and Asp141 from the C-subunit and Asn259 from the A-subunit in SBMV to be superposed respectively on Asp183, Asp186 and Asp225 from the corresponding subunits in TBSV. Residues Asp138 and Asp141 in SBMV are equivalent by structural-based sequence alignment to residues Asp183 and Asp186, respectively, in TBSV; the C-terminal residue Leu260 in SBMV corresponds to the start of the P-domain in TBSV.
